# Common threads in cardiac fibrosis, infarct scar formation, and wound healing

**DOI:** 10.1186/1755-1536-5-19

**Published:** 2012-11-01

**Authors:** Michael P Czubryt

**Affiliations:** 1 R4008 St. Boniface Research Centre, 351 Tache Avenue, Winnipeg, Manitoba, R2H 2A6, Canada

**Keywords:** Cardiac infarct scar, Collagen, Fibrosis, Wound healing

## Abstract

Wound healing, cardiac fibrosis, and infarct scar development, while possessing distinct features, share a number of key functional similarities, including extracellular matrix synthesis and remodeling by fibroblasts and myofibroblasts. Understanding the underlying mechanisms that are common to these processes may suggest novel therapeutic approaches for pathologic situations such as fibrosis, or defective wound healing such as hypertrophic scarring or keloid formation. This manuscript will briefly review the major steps of wound healing, and will contrast this process with how cardiac infarct scar formation or interstitial fibrosis occurs. The feasibility of targeting common pro-fibrotic growth factor signaling pathways will be discussed. Finally, the potential exploitation of novel regulators of wound healing and fibrosis (ski and scleraxis), will be examined.

## Introduction

Far from being merely an inert supporting scaffold, the cardiac extracellular matrix (ECM) is a dynamic structure that is in constant two-way communication with its embedded cells, such as myocytes and fibroblasts. Physical forces are integrated and transmitted by the ECM to these cells via cell–matrix interactions, resulting in activation of intracellular signaling pathways that both alter cell function and feed forward to induce changes in ECM structure via the release of matrix components or remodeling enzymes [1]. Information on the physical condition of the ECM is also encoded in the release of matrix-bound growth hormones or ECM constituents such as matrikines, providing another layer of complexity to the interaction of the ECM with its underlying cells.

In response to injury such as myocardial infarction, the heart undergoes a wound-healing process that shows remarkable parallels with other wound-repair processes such as that occurring in the skin after physical trauma, despite the significant differences in the basic nature of these disparate tissues [2]. Fibrosis occurs when ECM synthesis outpaces degradation, and is a common pathological outcome in both the skin and the heart. A deeper understanding of wound healing and fibrosis may be obtained by examining these processes in both tissues, revealing the potential for mechanisms, pathways and possibly even therapies common to both.

## Wound healing

**Figure 1:**
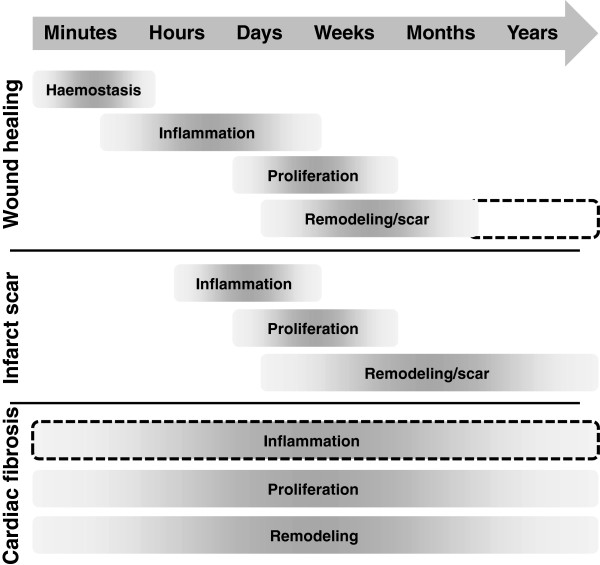
**Time course of phases of wound healing, infarct scar formation, and cardiac fibrosis.** The four phases of wound healing are hemostasis, inflammation, cell proliferation (including of fibroblasts), and ECM synthesis/remodeling and scar formation. The precise timing of these events is variable (indicated by the grayscale gradients), depending on the severity of the wound and the presence of exacerbating factors (for example, infection). Ideally, wounds will heal fully, but scars may persist for many years or the life of the individual (dashed lines). Infarct scar formation begins with removal of dead cells (not shown) and is followed by similar phases of inflammation, proliferation, and scar formation/remodeling. Unlike in wound healing, myofibroblasts may persist in the scar for years, leading to long-term remodeling. In interstitial cardiac fibrosis, the precise timing of the initiating event may be impossible to determine, and the phases of cell proliferation and ECM remodeling may continue over spans of years. An inflammatory component may also be present (dashed box), depending on the nature of the underlying insult.

Following acute injury, wounded tissue undergoes a series of four stages aimed at repairing the injury and returning the tissue, as much as possible, to the pre-injured state (Figure 1) [3]. The mechanism of dermal wound healing is arguably the best understood at present, and is the focus of this section. The first stage of the healing process is hemostasis, consisting of coagulation and platelet activation at the site of injury. These events serve to rapidly stem blood loss by the formation of a fibrin clot, and occur shortly after the injury (typically within minutes). The coagulation process is well-characterized; a full description of the various factors and mechanisms involved is beyond the scope of this review, and the reader is directed to recent publications on this subject [4,5].

Cytokines and growth factors (for example, transforming growth factor (TGF)-β) released by platelets also contribute to the proliferation and/or recruitment to the injury site of cells involved in the second wound-healing stage, inflammation, which can last for several days. These cells include neutrophils, monocytes/macrophages, and lymphocytes. Neutrophils provide the first line of attack against bacteria that may have entered the wound, and are recruited early in the inflammation process. Monocytes and macrophages arrive later in this stage, and perform several key functions, including further destruction and phagocytosis of bacteria, removal of necrotic tissue, and secretion of growth factors such as TGF-β, fibroblast growth factors (FGFs), and platelet-derived growth factor (PDGF), to induce fibroblast proliferation or recruitment to the injury site.

Even as the inflammation phase begins to decrease, the proliferative phase begins, and this lasts for days to weeks. As fibroblasts enter the injured region in response to growth factors such as PDGF, they proliferate and undergo myofibroblast conversion. Myofibroblasts are a highly synthetic derivative of fibroblasts or other cell types, capable of generating significantly larger amounts of ECM, and owing to their increased expression of α-smooth muscle actin, are also contractile. Although conventional wisdom holds that myofibroblasts arise by *in situ* phenoconversion of existing fibroblasts, other sources such as recruitment of circulating progenitors or epithelial-to-mesenchymal transition of local precursors have been reported, although the relative contribution of each remains unclear, and probably varies between tissues [6]. As myofibroblasts accumulate in the injured region, they begin to synthesize significant amounts of ECM, which begins the fourth and final phase, that of remodeling and scar formation, which can persist for months and initially overlaps with the proliferative phase.

The proliferative phase is marked by increasing numbers of fibroblasts, angiogenesis to restore tissue perfusion, formation of granulation tissue, and re-epithelialization, as epidermal epithelial cells migrate inwards from the wound periphery. Thus, the early hemostasis and inflammatory phases serve to attenuate blood loss and to clean and debride the injury site in preparation for the influx of cells needed to rebuild the tissue. During the proliferative phase, fibroblasts synthesize ECM components, including glycoproteins such as fibronectin, proteoglycans such as heparan sulfate, and fibrillar collagens, including types I and III, which predominate in the ECM. During the re-epithelialization process, non-fibrillar collagens (for example, type IV collagen) are also synthesized as part of the newly constructed basement membrane. This initial matrix formation helps to physically build up the wounded area and provides structural integrity.

Eventually the synthesized ECM undergoes extensive remodeling over several weeks (with the length of time depending in part on the size of the injury). Matrix synthesis continues, while at the same time, degradation of the ECM occurs as a result of the release of matrix metalloproteinases (MMPs) from fibroblasts and remnant inflammatory cells. These concurrent processes effectively remodel the tissue and mature the scar. Type III collagen is produced in greater amounts during early ECM formation, but is gradually degraded and replaced by type I collagen to increase the strength of the repair, which is further enhanced by collagen cross-linking. The activity of MMPs is fine-tuned by the concurrent release of the tissue inhibitors of MMPs (TIMPs). At the same time, the wounded area is kept under tension via the action of fibroblasts/myofibroblasts. This contraction reduces the wound area and hastens the healing process.

Two models of wound contraction have been proposed to explain how tension is generated, both of which rely on cell–ECM interactions. The first model posits that locomotion of fibroblasts in contact with other cells and ECM results in tension generation. This theory is supported by studies on collagen lattice contraction, which have shown that fibroblasts alone were sufficient to exert tractional forces [7,8]. The myofibroblast model, by contrast, proposes that myofibroblasts, which synthesize much greater amounts of the contractile protein, α-smooth muscle actin, than do fibroblasts, are responsible for tension generation by actively contracting to generate force [9]. There are data to support both models, and it remains unclear which is correct, or even if one model or the other may predominate in specific biologic settings [10]. Correct tension development is crucial for healing to proceed properly: insufficient tension may impede the healing process, or even lead to chronic wounds, whereas excessive tension may contribute to scarring.

With time, the injured tissue regains as much as 80% of its original strength, and healing is completed. Remnant myofibroblasts are removed by apoptosis, and a similar fate may occur to blood vessels that were formed to aid in repair [11]. Ideally, wound healing results in fully repaired tissue. The fetus is capable of such ideal healing, resulting in scar-free tissue repair. However, this capability seems to be lost with age, and the likelihood of defective wound healing increases significantly. The mechanisms underlying this shift are not fully understood.

## Defective wound healing

Given the complexity and typically extended timeframe over which the wound-healing process occurs, it is not surprising that defects in healing can occur. These defects generally fall into one of two categories: chronic wounds (for example, ulcerous lesions), in which the healing process is delayed, blocked, or otherwise interfered with, and excessive wound healing (for example, hypertrophic scars, keloids), in which the repair process does not attenuate properly or is ‘hyperactivated.’

Chronic wounds represent a significant clinical concern. Wound healing is typically impaired in diabetes mellitus, and chronic foot ulcers are a common complication of this disease [12]. A large number of factors can negatively affect the wound-healing process, leading to chronic wounds. The continued presence of foreign bodies or infectious agents can significantly extend the inflammatory phase, leading to failure of wound healing. Defects in wound perfusion or drainage also contribute to the formation of chronic wounds. A recent study by Wall *et al*. demonstrated that fibroblasts isolated from chronic wounds exhibit defects in proliferation, susceptibility to oxidative stress, premature senescence, and reduced chemokine expression compared with healthy fibroblasts [13]. This result suggests that alterations in normal fibroblast function may contribute to the pathogenesis of chronic wounds, although it is unclear why fibroblast function is compromised. Decreases in cell function with age may also play a role; as noted above, fetal tissue exhibits better wound healing than older tissue. Differences in gene expression, as determined by microarray analysis, have been reported for fetal versus aged skin [14].

During the proliferative and remodeling phases of wound healing, ECM synthesis initially outpaces ECM degradation. Eventually, however, a steady state is reached between these processes, coinciding with scar maturation. Excessive wound healing occurs when ECM synthesis remains high for longer than normal, resulting in overproduction of collagen and other ECM components. This condition may arise from a failure of myofibroblasts to undergo apoptosis and/or senescence to resolve the healing process, and results in hypertrophic scarring, in which the site of healing is marked by a raised scar [15]. A related condition is dermal keloid formation, which is characterized by significant overproduction of type III or type I collagen, respectively, during the early and late phases of keloid production. In contrast to hypertrophic scars, keloids extend beyond the boundaries of the original injury, and typically, the size of the keloid is disproportionate to the size of the initial wound. The underlying causes of keloids remain unknown, and the success rate of treatments varies. For example, surgical removal of keloids without adjunctive therapy (such as, corticosteroids) is of limited efficacy, because the chance of the keloid recurring can be greater than 50%, but newer approaches including laser therapy have produced better long-term recovery [16,17].

Recently, it has been realized that fibroblasts can behave as immune modulators by releasing various cytokines and chemokines, which in turn alter immune cell homing [18]. Because fibroblasts are a heterogeneous population, the nature of these released substances varies by tissue type, and thus can have differential effects on inflammatory responses [19]. Inappropriate persistence of fibroblasts or myofibroblasts in the healing wound may thus not only exacerbate ECM production, but may also result in prolonged inflammation, which further contributes to hypertrophic scar formation [20].

## Myocardial infarction, repair, and fibrosis

Cardiac muscle requires a continual source of nutrients and oxygen to support the prodigious workload of the heart, which beats more than 100 000 times per day. The coronary arteries efficiently perfuse the cardiac muscle, but atherosclerosis can result in blockage of these arteries via plaque formation or thromboembolism, starving the downstream myocardium of oxygen. If this situation is not resolved quickly, rampant tissue death occurs, and a large infarct zone is created. This region must be repaired quickly because mechanical failure of the infarcted tissue can result in aneurysm owing to the high pressures generated in the contracting ventricles. Cardiac myocytes are largely terminally differentiated and have exited the cell cycle, thus proliferation of myocytes is not a viable process for repair of the myocardium. Instead, the heart undergoes a repair process that exhibits a number of similarities to dermal wound healing.

After an infarct occurs, various inflammatory cells migrate to the damaged region, a process that is similar to the inflammatory phase of wound healing (Figure 1) [2]. Removal of dead and dying tissue ensues, and the release of growth factors and cytokines from inflammatory cells such as monocytes and macrophages attracts to the site of injury cells that are destined to become myofibroblasts. The conventional model has been that local fibroblasts migrate to the damaged region, where they convert to myofibroblasts and begin to synthesize large amounts of ECM to effect repair [21]. Although fibroblasts are much smaller than cardiomyocytes, they are more numerous, which is probably crucial to the ability of the heart to repair itself after infarction.

The source of these myofibroblasts is controversial; although the myocardium itself is certainly a significant contributor of cells, recent evidence has implicated the recruitment of circulating fibrocytes or stem cells that can trans-differentiate to myofibroblasts, and has also implicated the process of epithelial-to-mesenchymal transition [22,23]. Regardless of the source, ECM synthesis is dramatically increased within days of injury, similar to the proliferative phase of wound healing, and this is indicative of a beneficial ‘reparative’ fibrosis.

Over several weeks, collagen fibers are constructed throughout the infarct zone; these are initially composed primarily of type III collagen, but are eventually replaced with type I collagen. Maturation of collagen fibers, including significant cross-linking, continues to increase the tensile strength of the developing scar. Again similar to wound healing, the scar undergoes extensive remodeling as various MMPs and TIMPs are secreted and process the ECM. Within 8 weeks of injury, the scar is fully mature.

Although the similarities between cardiac and dermal wound healing are striking, there are also several crucial differences. The most obvious is the fate of the scar itself. In dermal wound healing, the scar ECM typically regresses over time as the injured area is recellularized, and except in the case of defective healing, the scar volume is eventually greatly minimized, to the point that the scar may not be visually obvious. In the heart, however, cardiomyocytes are terminally differentiated and have left the cell cycle, thus they fail to repopulate the scar in sufficient numbers to effect repair, resulting in a scar that persists for the life of the patient. Furthermore, whereas myofibroblasts in dermal wound healing are progressively removed by apoptosis, myofibroblasts in the cardiac infarct scar can persist for many years [24]. This can have untoward effects on cardiac function; besides impairing cardiac contraction and relaxation, both myofibroblasts and the ECM they produce exhibit electrical properties different from those of the surrounding tissue, contributing to arrhythmogenesis [25]. Thus, although the initial overall healing process is very similar between the heart and other injured tissues, the end result is very distinct, owing to the unique cellular make-up of the myocardium.

Another important difference between healing in the heart and that in other tissues is that, for reasons that are not fully understood, distal regions of the heart, not directly involved in the initial infarct event, typically undergo a gradual ‘reactive’ fibrotic process as diffuse ECM synthesis proceeds (Figure 1). This distal fibrosis has important clinical ramifications; as the myocardium becomes progressively stiffer, both contraction (expulsion of blood) and relaxation (refilling of the ventricles) becomes impaired. The long-term result is a reduction of cardiac output, which, if sufficiently severe, manifests in cardiac failure, increasing the morbidity and likelihood of mortality of the patient. Regions of fibrosis also exhibit altered conduction characteristics and thus may contribute to arrhythmia generation, while fibroblasts themselves exhibit different electrical features from those of cardiomyocytes, which may provide an additional mechanism for arrhythmogenesis [26].

Although fibrosis occurring after infarction is an important clinical problem, it is noteworthy that cardiac fibrosis results even more commonly from other stresses on the heart, including congenital defects, hypertension, or dilated cardiomyopathy [27]. Various theories have been advanced for how fibrosis begins, including altered tension/stress in the myocardium, activation of TGF-β signaling, or inappropriate activation of fibroblast to myofibroblast conversion [28]. Hypoxia has also been proposed to be pro-fibrotic in the heart via induction of hypoxia-inducible factor (HIF)-1α, which may explain the increased fibrosis in cardiac allograft remodeling, because graft perfusion may be suboptimal [29]. This parallels the situation in dermal wound healing, where hypoxia-induced HIF-1α upregulation results in increased ECM production [30]. Although hypoxia can thus promote the healing process, excessive or prolonged hypoxia may be detrimental, and it was recently shown that hypoxia may contribute to exuberant granulation tissue fibrosis, an equine wound-healing disorder that resembles human keloids [31].

Fibrosis *in vivo* is probably the result of a complex interplay between multiple factors such as those described above. The nature of the fibrosis can also vary, from focal fibrotic lesions to diffuse patches of fibrosis, as can the distribution of the fibrosis in the heart, depending on the underlying pathological condition; for example, dilated cardiomyopathy exhibits a high prevalence of left atrial fibrosis [32]. However, it remains unclear whether the underlying mechanisms driving fibrosis in these various cases are the same as those contributing to infarct scar formation or post-infarct interstitial fibrosis. There is evidence that the myofibroblasts that contribute to scar formation may arise from different cellular precursors than those that contribute to diffuse fibrosis; that is, resident cardiac cells (for example, fibroblasts or mesenchymal stem-like cells) in the former case, versus myeloid circulating cells (for example, monocytes) in the latter [22,33]. These fundamental disparities may thus result in a continuum of conditions that may collectively be called ‘fibrosis’, yet may arise through divergent mechanisms.

## Potential therapeutic targets in fibrosis and wound healing

Activation of fibroblasts to myofibroblasts and subsequent induction of ECM and collagen synthesis are common phenomena in wound healing, hypertrophic scar development, infarct scar formation, and cardiac interstitial fibrosis. These processes are thus a double-edged sword; they are crucial for proper wound-healing or infarct scar formation to occur, but inappropriate activation of these processes results in pathologic functional impairment. Emerging evidence indicates that common molecular mechanisms may underlie both the reparative and pathological aspects of wound healing and fibrosis. In particular, a number of growth factors, including TGF-β, insulin-like growth factor (IGF)-1, and connective tissue growth factor (CTGF) play stimulatory roles in these processes.

TGF-β behaves as a fibroblast mitogen in the early stages of wound healing, promoting fibroblast to myofibroblast conversion, and directly upregulating collagen synthesis via activation of the Smad signaling pathway. Smads such as Smad3 directly bind to and transactivate collagen gene promoters, and play key roles in cardiac infarct scar formation [34-36]. Importantly, TGF-β plays similar roles in wound healing, cardiac infarct scar formation, and cardiac fibrosis. Indeed, TGF-β has been implicated in fibrosis of multiple tissue types including liver, kidney, and airway [37-39].

It is thus not surprising that multiple therapeutic strategies targeting TGF-β for the attenuation of fibrosis have been proposed and tested with some degree of success. However, given the tremendous variety of roles played by TGF-β across many cellular processes and tissue types, caution must be exercised in this approach. For example, blockade of TGF-β has shown great promise for targeting a variety of tumors. At the same time, however, TGF-β exerts a number of tumor-suppressor effects, and thus targeting of this pathway may actually exacerbate the formation of a subset of malignancies [40]. Evidence of the pleiotropy of this pathway is also seen after genetic deletion of Smad3 in mice. Smad3 null mice exhibit improved and more rapid healing of deep tissue wounds, possibly as a result of reduced inflammatory cell infiltration [41], but conversely, Smad3 deletion impairs cardiac infarct scar formation, owing to impaired fibroblast function [36]. Targeting of fibrosis via TGF-β must therefore be carefully considered in the context of the whole patient, with emphasis on minimizing deleterious off-target effects.

Like TGF-β, IGF-1 has been shown to play roles in both wound healing and fibroblast function. Exogenous recombinant human IGF-1 administered onto full-thickness wounds in diabetic db/db mice significantly accelerated healing and capillary density at the site of injury [42]. IGF-1 expression increases sharply by 3 days after dermal wounding in healthy animals, but basal expression of IGF-1 is reduced in diabetic mice, and is not induced in diabetic skin wounds until 14 days after injury, with peak expression further delayed to 21 days [43]. The number of IGF-1-expressing cells is significantly higher in dermal hypertrophic scars after burn damage compared with normal skin from the same patients [44]. IGF-1 expression has also been reported to be decreased both in skin and in healing foot ulcers in humans, and IGF-1 resistance is associated with impaired wound healing in diabetic rats [45,46]. IGF-1 levels thus seem to be proportional to the degree to which the wound-healing process is activated, being low or delayed in chronic wounds (for example, in diabetes) compared with healthy tissue, and relatively high during normal healing or hypertrophic scarring.

IGF-1 acts as a potent mitogen for airway fibroblasts [47]. Macrophages stimulated by interleukin (IL)-4 release IGF-1, which in turn attenuates lung myofibroblast apoptosis after withdrawal of growth factor [48]. Although the specific role of IGF-1 in cardiac fibroblasts has been less studied, it has been found that IGF-1 stimulates type I collagen synthesis in these cells [49]. Kanellakis *et al*. recently showed that macrophage-derived IL-4 is pro-fibrotic in the heart, although the specific role of IGF-1 was not identified in this study [50]. IGF-1 was also shown to reduce the death rate of isolated cardiac fibroblasts after simulated ischemia/reperfusion injury by attenuating apoptosis [51].

Given these observations, it is tempting to speculate that targeting of IGF-1 in fibrosis may be therapeutically useful. However, IGF-1 also demonstrates beneficial effects in other tissues, most notably cardiomyocytes, in which a precise balance of IGF-1 expression is crucial for normal cell function. Whereas overexpression of IGF-1 may contribute to acromegaly, moderate increases in IGF-1 expression seem to be important for physiological hypertrophy of the heart [52]. The need for a precise balance in IGF-1 expression was further shown by a report that long-term IGF-1 expression in a transgenic mouse model induced physiologic hypertrophy in the short term, followed by pathologic hypertrophy and fibrosis in the long term [53]. IGF-1 also exerts anti-apoptotic effects on cardiomyocytes (just as it does in myofibroblasts) [54,55]. Targeting IGF-1 as part of an anti-fibrotic strategy is thus likely to have marked negative effects on cardiac function.

Other growth factors such as CTGF, basic (b)FGF, and angiotensin II have been implicated in wound healing and fibrosis in a variety of tissues [56-59]. However, strategies to inhibit these factors are fraught with difficulties similar to those associated with TGF-β or IGF-1, again because of the pleiotropic effects of such molecules. CTGF plays a central role in inducing fibrosis by acting downstream of TGF-β, but it also behaves as a pro-angiogenic and chondrogenic factor [60-62]. bFGF regulates apoptosis of myofibroblasts but not fibroblasts, and thus may be important in the final stages of wound healing; however, it also exhibits various cardioprotective effects [58,63,64]. Angiotensin II promotes dermal wound healing, but also exerts potent effects on blood pressure [59]. Thus, the general strategy of targeting regulatory growth factors is unlikely to be therapeutically feasible given the many disparate roles that such factors typically play. It is therefore crucial to identify novel regulators of fibrosis for potential anti-fibrotic strategies.

## New therapeutic targets: ski and scleraxis

The proto-oncogene ski interferes with TGF-β-mediated signaling by binding directly to Smads, blocking their downstream effects on gene expression [65]. In dermal fibroblasts, Smad3 inhibits cell proliferation, but this effect is reversed upon overexpression of ski [66]. In the same study, it was shown that ski expression peaks approximately 9 days after injury in a full-thickness dermal wound-healing model. Ski was reported to increase fibroblast proliferation, while at the same time attenuating apoptosis. In a later study, the same group reported that high concentrations of TGF-β, which inhibits skin fibroblast proliferation, decreased ski expression, whereas low concentrations, which induce fibroblast proliferation, resulted in increased ski expression [67]. Knockdown of ski blocked the biphasic effect of TGF-β on proliferation, suggesting that the mechanism of this effect is dependent on ski. Finally, this group also reported that although ski promoted fibroblast proliferation, it also decreased type I collagen synthesis [68]. Because knockout of Smad3 is associated with accelerated wound healing, increasing ski expression or activity may provide a means to positively regulate the healing response [41].

Intriguingly, it was recently reported that ski may play a similar role in the heart. Overexpression of ski in isolated cardiac myofibroblasts reduced type I collagen expression and myofibroblast contractility [69]. This latter effect may be due to an observed decrease in expression of α-smooth muscle actin in ski-infected cells. The authors proposed that ski may act to ‘regress’ the myofibroblast phenotype back to that of a fibroblast; however, they also noted that, unlike the situation in dermal fibroblasts, ski overexpression increased myofibroblast apoptosis. Thus, in the heart, augmenting ski expression or function may both decrease fibrosis and reduce myofibroblast numbers, which may be clinically useful in interstitial fibrosis. The E3 ubiquitin ligase Arkadia targets negative regulators of TGF-β signaling, including Smad7 and ski, for degradation [70], thus a possible approach to increasing ski function may be to inhibit the action of Arkadia.

The basic helix-loop-helix transcription factor scleraxis was originally cloned in a screen for novel E47-interacting partners in the heart [71]. Scleraxis is a developmental marker for a variety of collagen-rich tissues such as tendons and cardiac valves, and genetic deletion of scleraxis results in numerous defects in tendon formation [72]. We recently reported that scleraxis directly transactivates the human collagen Iα2 gene promoter, and that scleraxis overexpression is sufficient to increase collagen Iα2 expression in primary cardiac myofibroblasts [73]. We also found that cardiac fibroblast scleraxis expression increases in response to TGF-β or after conversion of fibroblasts to myofibroblasts. Importantly, scleraxis expression also increased nearly four-fold in the infarct scar after surgical coronary artery ligation in the rat, similar to the expression of collagen Iα2. Others have reported that scleraxis regulates collagen Iα1 gene expression in tenocytes [74]. We generated a scleraxis DNA-binding mutant that dose-dependently interfered with transactivation of the collagen Iα2 gene promoter, further suggesting that scleraxis is a key regulator of collagen synthesis [73]. Importantly, this mutant was able to completely attenuate both basal and TGF-β-induced collagen production in primary cardiac fibroblasts [75].

**Figure 2:**
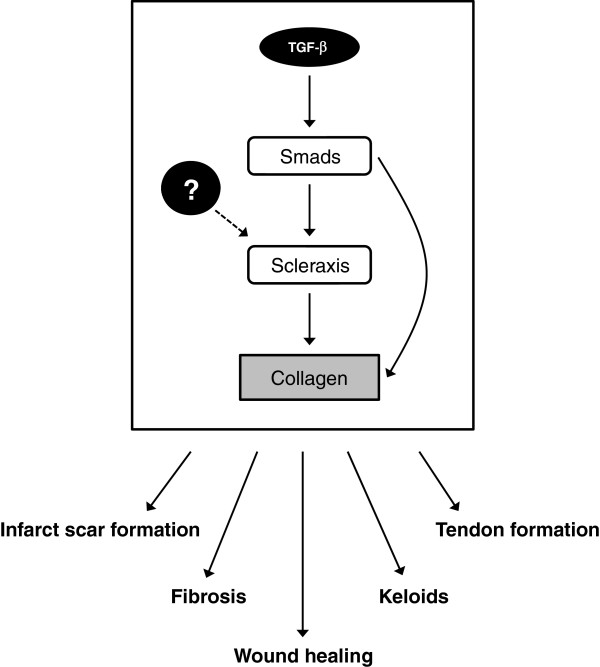
**Mechanism of collagen gene regulation by scleraxis.** Scleraxis expression is increased in response to transforming growth factor (TGF)-β via the canonical Smad signaling pathway [73,75]. Collagen synthesis is upregulated by scleraxis and/or by Smads (for example, Smad3), either independently or synergistically via direct interaction with the collagen gene promoter [75]. It is unclear whether other mechanisms may upregulate scleraxis expression independently of TGF-β (dashed line). These mechanisms may act as a regulatory ‘cassette’, governing cardiac infarct scar formation [73], cardiac fibrosis (and potentially fibrosis in other tissues as well) [73,75], tendon formation [72,77], and possibly keloid formation [78] and wound healing [79]. Therapeutic attenuation of scleraxis expression or activity may provide a means to alter one or more of these processes.

Given these various results, we hypothesized that scleraxis may regulate fibrillar collagen gene expression not only in the heart, but probably in other tissues as well, by acting as a conserved pro-fibrotic regulator (Figure 2). As clearly shown by Murchison *et al*., scleraxis is crucial in tendon development, and it is striking that the affected tendons also showed a dramatic loss of type I collagen expression [72]. Our data also provide evidence that scleraxis is a regulator of type I collagen expression in cardiac fibroblasts and myofibroblasts downstream of TGF-β. Preliminary data from our laboratory indicates that scleraxis is expressed in airway smooth muscle cells, which synthesize fibrillar collagens in the lungs (data not shown). It has also been recently shown that TGF-β augments expression of both collagen Iα2 and scleraxis in skeletal muscle [76]. It is therefore tempting to speculate that scleraxis behaves as a regulator of fibrillar collagen synthesis across multiple tissue types. A corollary to this hypothesis is that fibrosis may arise when scleraxis function or expression increases inappropriately.

A role for scleraxis in wound healing has not yet been identified. However, it has been shown that, whereas scleraxis is not expressed by healthy dermal fibroblasts, it is strongly upregulated in fibroblasts isolated from dermal keloids, which are composed primarily of fibrillar collagens I and/or III [78]. Given our finding that scleraxis is also upregulated in the healing cardiac infarct scar, the ability of scleraxis to regulate type I collagen synthesis may contribute to the remodeling and scar-formation phases of the wound-healing process. Whether long-term overexpression of scleraxis results in inappropriate collagen production and/or fibrosis (for example, in keloids or hypertrophic scars) remains to be determined.

Alberton *et al*. recently reported that overexpression of scleraxis in human bone marrow-derived mesenchymal stem cells appeared to induce a tendon progenitor cell fate, including increased collagen I expression [77]. Scleraxis may thus be a master regulator for tenocytes, and regulation of fibrillar collagen synthesis may represent one aspect of this role. Ultimately, fibrillar collagen production could be governed by a gene program that is conserved across tissues and during the wound-healing process. Growing evidence suggests that scleraxis is a central player in this conserved gene program. As noted above, gene deletion of scleraxis resulted in reduced collagen I in tendons, whereas overexpression of scleraxis was shown to induce collagen I expression in pluripotent tenocyte precursors, tenocytes, and cardiac fibroblasts [72,74,77]. Modulation of scleraxis function may therefore provide a means to fine-tune the production of fibrillar collagens, using attenuation of scleraxis (for example, by small molecule inhibitors) to reduce keloid formation or fibrosis in multiple tissue types, and augmentation of scleraxis (for example, by transgene delivery) to improve infarct scar formation or tissue strength (such as in tissue grafts or on formed tissue scaffolds) by augmenting fibrillar collagen production. Given the failure of previous attempts to target fibrosis, coupled with the likelihood that treatments aimed at growth factors will fail because of off-target effects, scleraxis should be evaluated in the short term as a possible target for therapeutic drug design.

Transcriptional regulators such as ski and scleraxis have traditionally been considered to be ‘undruggable’; they lack active sites, possess no pore or channel to be blocked, and typically do not have deep surface involutions suitable for binding of small molecules, thus the usual approaches to inhibitor design are inapplicable. Recently, however, proof of concept has been shown for a strategy to target transcriptional regulators using hydrocarbon-stapled peptides. Using this approach, Moellering *et al*. demonstrated inhibition of the NOTCH transcription-factor complex using an engineered peptide mimicking a dominant-negative NOTCH-interacting region of Mastermind-like (MAML)1, which effectively prevented formation of a functional transcriptional complex [80]. This inhibitor peptide repressed NOTCH target gene expression and blocked proliferation of T-cell acute lymphoblastic leukemia cells, in which NOTCH is inappropriately activated.

Stapled peptides may similarly represent a useful strategy to regulate ski and scleraxis function therapeutically. A stapled peptide designed to mimic the region of ski that interacts with Smads may be effective in also mimicking the function of ski, resulting in repression of the pro-fibrotic gene expression program. With respect to scleraxis, it is unclear at present exactly how our dominant-negative mutant represses collagen gene expression. The mutant lacks a DNA-binding domain, yet retains its protein-interaction domain, thus we hypothesize that the mutant may sequester crucial transcriptional partners to block expression of gene targets, similar to the Inhibitor of Differentiation proteins such as Id2 [73,75]. Given this scenario, a stapled peptide designed to mimic the scleraxis protein-interaction domain may recapitulate the repressive function of the mutant to provide effective anti-fibrotic activity.

Such peptide-based approaches may be broadly applicable to fibrosis of various tissues and organs; however, it is important to carefully consider the timing of any such treatment. For example, after myocardial infarction it would probably be detrimental to provide an anti-fibrotic immediately, as this would be likely to interfere with the normal formation of the infarct scar. However, treatment at later times, after scar maturation has completed, may then provide a therapeutic benefit against interstitial fibrosis. It also remains to be seen whether such treatment would be effective in patients with pre-existing fibrosis. Because collagen and other ECM components regularly turn over as a result of constant degradation and synthesis, a reduction in net collagen synthesis may be beneficial even when fibrosis is advanced [81]. Another issue to be overcome is the targeting of collagen turnover specifically in the heart; because turnover rates in the heart seem to be somewhat higher than in other tissues such as the skin, it is possible that anti-fibrotic treatments would have a greater impact in the heart than elsewhere [81,82], reducing off-target effects.

## Common threads

Fibroblasts, collectively, are a heterogeneous cell type, reflecting their different roles in different tissue types. This heterogeneity forms, for example, the basis of a putative ‘stromal address code’; differential expression of fibroblast cell-surface proteins and secreted cytokines results in differential recruitment of leukocytes, in turn governing the nature of the inflammatory responses in different tissues [18]. Yet, despite this heterogeneity, fibroblasts generally play similar roles regardless of their anatomical location, including regulating the synthesis and degradation of ECM and playing active roles in wound healing.

There are certainly differences in dermal versus cardiac wound healing, including the timing and duration of the various stages (Figure 1). Arguably, the most crucial difference is the recellularization of dermal wounds, which hastens healing and results in more or less complete injury recovery, whereas at the same time excessive ECM is degraded to reduce scarring. By contrast, cardiac injury (that is, infarction) does not completely resolve, because cardiomyocytes fail to repopulate the infarcted region and limited recellularization occurs via recruitment of myofibroblasts. Excess ECM remains for the life of the patient, although remodeling may continue for long periods of time.

In spite of these differences, the common threads between dermal and cardiac wound healing are numerous. Although the specific timing may be somewhat different, the major stages of wound healing (inflammation, proliferation, and remodeling) are effectively the same (Figure 1). Similarly, these stages are governed by the same basic cell types as noted above, with a central role for fibroblasts in both tissues. Many of the same intracellular signaling pathways and mechanisms are also involved in both processes, including TGF-β and Smads, as well as HIF-1α in the presence of hypoxia. Although a specific role for scleraxis in wound healing in both tissues remains to be definitively demonstrated, the finding that scleraxis expression is greatly upregulated in both dermal keloids and the cardiac infarct scar indicates that greater scrutiny of its role is required [73,78]. Recently, it was reported that scleraxis is also upregulated in murine patellar tendon injuries, further suggesting that scleraxis may have a central role in injury repair [79]. Indeed, as scleraxis has been shown to be capable of driving mesenchymal stem cells to a tenocyte fate, it has been proposed that scleraxis-overexpressing stem cells may be effective for improving healing of rotator cuff injuries by promoting ECM remodeling [77,83]. It is unknown at this time whether ski also participates in dermal wound healing; however, it has been shown to promote dermal fibroblast proliferation, while at the same time inhibiting collagen synthesis [68].

In contrast to infarct scar formation, the interstitial fibrosis that follows, or that arises in response to other conditions such as hypertension, exhibits multiple aspects of the wound-healing program contemporaneously (Figure 1); inflammation, proliferation and remodeling may all be occurring at the same time. Although interstitial fibrosis lacks the frank injury that demarks infarction or dermal wounds, it is tempting to consider this process in the context of wound healing gone awry, similar to the dysfunctional healing process in dermal keloids or hypertrophic scars. Each of these processes is characterized by overproduction of ECM components by myofibroblasts that are over-represented in the tissue (because of inappropriate activation of fibroblasts or other precursor cells, and/or by decreased apoptosis). Chronic inflammation and tissue hypoxia have been implicated in both processes. Finally, similar molecular signals are involved, including TGF-β, Smads, IGF-1, CTGF and angiotensin II.

Given these myriad similarities between dermal wound healing, cardiac infarct scar formation, and fibrosis of both the skin and the heart, it is likely that further insight into each of these processes can be obtained by examining the others, despite the differences that also exist. It is also possible that therapies targeting one of these processes may be effective in the others; for example, an anti-fibrotic developed for the heart may be useful for improper wound healing in the skin. Scleraxis and ski represent possible targets that should be considered for one or more of these processes, and further study may identify still more candidates for therapeutic intervention.

## Abbreviations

bFGF: Basic fibroblast growth factor; CTGF: Connective tissue growth factor; ECM: Extracellular matrix; FGF: Fibroblast growth factor; HIF-1α: Hypoxia-inducible factor 1α; IGF-1: Insulin-like growth factor 1; PDGF: Platelet-derived growth factor; TGF-β: Transforming growth factor β.

## Competing interests

The author declares that he has no competing interests.
